# Supplementary material for: Eating habits of students of health colleges and non-health colleges at the Northern Border University in the Kingdom of Saudi Arabia
Source: PLoS One. 2024 Oct 28;19(10):e0312750. doi: 10.1371/journal.pone.0312750 (PMC11516010; doi:10.1371/journal.pone.0312750)
Supplement: S1 File — (DOCX) [file pone.0312750.s001.docx]

**Supplementary Table 1: Comparisons of eating habits between students at health and non-health colleges**

| Eating habits | | Non-health colleges  (N.=240) | | Health colleges  (N.=240) | | X^2^ | P |
| --- | --- | --- | --- | --- | --- | --- | --- |
|  |  | N. | % | N. | % |  |  |
| Do you have regular meals? | No | 146 | 60.8 | 151 | 62.9 | 0.22 | 0.64 |
|  | Yes | 94 | 39.2 | 89 | 37.1 |  |  |
| Do you have daily breakfast? | No | 132 | 55.0 | 144 | 60.0 | 1.23 | 0.27 |
|  | Yes | 108 | 45.0 | 96 | 40.0 |  |  |
| Frequency of daily meals | Less than three meals | 133 | 55.4 | 123 | 51.2 | 0.84 | 0.36 |
|  | Three or more times | 107 | 44.6 | 117 | 48.7 |  |  |
| Frequency of having snacks per week | Less than three times | 131 | 54.6 | 125 | 52.1 | 0.30 | 0.58 |
|  | Three or more times | 109 | 45.4 | 115 | 47.9 |  |  |
| Weekly consumption of vegetables and legumes | Less than three times | 151 | 62.9 | 145 | 60.4 | 0.32 | 0.57 |
|  | Three or more times | 89 | 37.1 | 95 | 39.6 |  |  |
| Weekly consumption of fruits | Less than three times | 201 | 83.7 | 204 | 85.0 | 0.14 | 0.71 |
|  | Three or more times | 39 | 16.2 | 36 | 15.0 |  |  |
| Weekly consumption of fried food | Less than three times | 152 | 63.3 | 137 | 57.1 | 1.96 | 0.16 |
|  | Three or more times | 88 | 36.7 | 103 | 42.9 |  |  |
| Consumption of fast food | Often | 71 | 29.6 | 68 | 28.3 | 0.09 | 0.95 |
|  | Sometimes | 157 | 65.4 | 160 | 66.7 |  |  |
|  | Rarely | 12 | 5.0 | 12 | 5.0 |  |  |
| Meals with family and friends | Daily | 113 | 47.1 | 121 | 50.4 | 0.53 | 0.46 |
|  | Not daily | 127 | 52.9 | 119 | 49.6 |  |  |
| Water intake | Less than two liters per day | 177 | 73.7 | 172 | 71.7 | 0.26 | 0.61 |
|  | Two or more liters per day | 63 | 26.2 | 68 | 28.3 |  |  |
| Type of food consumed | Mainly meat | 15 | 6.2 | 20 | 8.3 | 7.65 | 0.05 |
|  | Mainly vegetables | 13 | 5.4 | 4 | 1.7 |  |  |
|  | Carbohydrate | 70 | 29.2 | 86 | 35.8 |  |  |
|  | Variety of food in balance | 142 | 59.2 | 130 | 54.2 |  |  |

X^2^: The Chi-square test; Statistical significance was considered at P<0.05

**Supplementary Table 2: Comparisons of eating-related psychological factors between students at health and non-health colleges**

| Psychological factors | | Non-health colleges  (N.=240) | | Health colleges  (N.=240) | | X^2^ | P |
| --- | --- | --- | --- | --- | --- | --- | --- |
|  |  | N. | % | N. | % |  |  |
| Do you eat because you are feeling lonely? | Often | 31 | 12.9 | 16 | 6.7 | 5.51 | 0.06 |
|  | Rarely | 123 | 51.2 | 127 | 52.9 |  |  |
|  | Sometimes | 86 | 35.8 | 97 | 40.4 |  |  |
| Do you feel completely out of control when it comes to food? | Often | 21 | 8.7 | 24 | 10.0 | 1.43 | 0.49 |
|  | Rarely | 133 | 55.4 | 143 | 59.6 |  |  |
|  | Sometimes | 86 | 35.8 | 73 | 30.4 |  |  |
| Do you eat so much until stomach hurts? | Often | 29 | 12.1 | 26 | 10.8 | 1.91 | 0.38 |
|  | Rarely | 125 | 52.1 | 140 | 58.3 |  |  |
|  | Sometimes | 86 | 35.8 | 74 | 30.8 |  |  |
| Do you eat because of feeling upset or nervous? | Often | 27 | 11.2 | 32 | 13.3 | 1.1 | 0.57 |
|  | Rarely | 124 | 51.7 | 129 | 53.7 |  |  |
|  | Sometimes | 89 | 37.1 | 79 | 32.9 |  |  |
| Do you eat because you are feeling bored? | Often | 40 | 16.7 | 48 | 20.0 | 2.57 | 0.28 |
|  | Rarely | 90 | 37.5 | 74 | 30.8 |  |  |
|  | Sometimes | 110 | 45.8 | 118 | 49.2 |  |  |
| Do you eat because you are feeling happy? | Often | 54 | 22.5 | 51 | 21.2 | 0.11 | 0.95 |
|  | Rarely | 71 | 29.6 | 72 | 30.0 |  |  |
|  | Sometimes | 115 | 47.9 | 117 | 48.7 |  |  |

X^2^: The Chi-square test; Statistical significance was considered at P<0.05
