# Supplementary material for: Eating habits of students of health colleges and non-health colleges at the Northern Border University in the Kingdom of Saudi Arabia
Source: PLoS One. 2024 Oct 28;19(10):e0312750. doi: 10.1371/journal.pone.0312750 (PMC11516010; doi:10.1371/journal.pone.0312750)
Supplement: Attachment — Submitted filename: PONE-D-24-23613_reviewer (With Comments).pdf [file pone.0312750.s003.pdf]

PLOS ONE

Eating Habits of Students of Health Colleges and Non-Health Colleges at the Northern Border University in the Kingdom of Saudi Arabia

--Manuscript Draft--

**Manuscript Number:**	PONE-D-24-23613
**Article Type:**	Research Article
**Full Title:**	Eating Habits of Students of Health Colleges and Non-Health Colleges at the Northern Border University in the Kingdom of Saudi Arabia
**Short Title:**	Eating habits of university students at NBU
**Corresponding Author:**	Hanaa El-Sayed Abdel Rahman Bayomy, Ph.D<br>Benha University Faculty of Medicine<br>Benha, EGYPT
**Keywords:**	Eating habits; university students; Health colleges; Non-health colleges; Psychological factors; Northern Saudi Arabia
**Abstract:**	Background<br><br>Eating habits are important determinants for good health. However, young adults who have recently transitioned to university life experience stress and lack of time, which are major risk factors for poor eating habits. This study aimed to determine and compare eating patterns between students at health and non-health colleges at Northern Border University (NBU), Saudi Arabia and to identify the relationship between eating patterns and sociodemographic and psychological characteristics of students.<br><br>Methods<br><br>This comparative cross-section study was conducted on 480 students equally from health and non-health colleges at NBU from March to April 2023. A pre-designed electronic questionnaire was used to collect data on students' sociodemographic characteristics, eating habits, and diet-related psychological factors. Statistical analyses were carried out using STATA/SE version 11.2 for Windows.<br><br>Results<br><br>Unhealthy eating habits were reported by 44.6% and 41.3% of students at health and non-health colleges, respectively. Most students had irregular meals, skipped breakfast, ate fast food frequently, and consumed insufficient amounts of vegetables, fruits, and water. Parental separation, living away from family predisposed to unhealthy eating habits. Being 21-23 years old, playing sports, and high body mass index were linked to healthy eating habits. Psychological factors such as overeating until the stomach hurts and eating to feel happy were associated with unhealthy eating habits.<br><br>Conclusion<br><br>Unhealthy eating habits were prevalent among students at NBU irrespective of the type of study. Thus, implementing initiatives to promote nutrition and healthy eating habits within the university environment is crucial for health promotion and well-being among students.
**Order of Authors:**	Hanaa El-Sayed Abdel Rahman Bayomy, Ph.D
	Shmoukh Mushref Alruwaili
	Razan Ibrahim Alsayer
	Nuof Khalid Alanazi
	Dana Ahmed Albalawi
	Khulud Hamed AlShammari

Powered by Editorial Manager® and ProduXion Manager® from Aries Systems Corporation

	Mariam Mahmoud Ibrahim Moussa
**Additional Information:**	
**Question**	**Response**
**Financial Disclosure**<br><br>Enter a financial disclosure statement that describes the sources of funding for the work included in this submission. Review the submission guidelines for detailed requirements. View published research articles from *PLOS ONE* for specific examples.<br><br>This statement is required for submission and **will appear in the published article** if the submission is accepted. Please make sure it is accurate.<br><br>**Funded studies**<br>Enter a statement with the following details:<br>• Initials of the authors who received each award<br>• Grant numbers awarded to each author<br>• The full name of each funder<br>• URL of each funder website<br>• Did the sponsors or funders play any role in the study design, data collection and analysis, decision to publish, or preparation of the manuscript?<br><br>Did you receive funding for this work?	The author(s) received no specific funding for this work.
**Competing Interests**<br><br>Use the instructions below to enter a competing interest statement for this submission. On behalf of all authors, disclose any competing interests that could be perceived to bias this work—acknowledging all financial support and any other relevant financial or non-financial competing interests.<br><br>This statement is required for submission and **will appear in the published article** if the submission is accepted. Please make sure it is accurate and that any funding	The authors have declared that no competing interests exist.

sources listed in your Funding Information later in the submission form are also declared in your Financial Disclosure statement.<br><br>View published research articles from *PLOS ONE* for specific examples.<br><br>**NO authors have competing interests**<br><br>Enter: *The authors have declared that no competing interests exist.*<br><br>**Authors with competing interests**<br><br>Enter competing interest details beginning with this statement:<br><br>*I have read the journal's policy and the authors of this manuscript have the following competing interests: [insert competing interests here]*<br><br>* typeset	
**Ethics Statement**<br><br>Enter an ethics statement for this submission. This statement is required if the study involved:<br><br>• Human participants<br>• Human specimens or tissue<br>• Vertebrate animals or cephalopods<br>• Vertebrate embryos or tissues<br>• Field research<br><br>Write "N/A" if the submission does not require an ethics statement.<br><br>General guidance is provided below. Consult the submission guidelines for detailed instructions. **Make sure that all information entered here is included in the Methods section of the manuscript.**	This study was approved by the Local Committee of Bioethics (HAP-09-A-043) at Northern Border University no. (27/44/H). Written informed consent was obtained from all students prior to their participation.

Powered by Editorial Manager® and ProduXion Manager® from Aries Systems Corporation

**Format for specific study types**<br><br>**Human Subject Research (involving human participants and/or tissue)**<br>• Give the name of the institutional review board or ethics committee that approved the study<br>• Include the approval number and/or a statement indicating approval of this research<br>• Indicate the form of consent obtained (written/oral) or the reason that consent was not obtained (e.g. the data were analyzed anonymously)<br><br>**Animal Research (involving vertebrate animals, embryos or tissues)**<br>• Provide the name of the Institutional Animal Care and Use Committee (IACUC) or other relevant ethics board that reviewed the study protocol, and indicate whether they approved this research or granted a formal waiver of ethical approval<br>• Include an approval number if one was obtained<br>• If the study involved *non-human primates*, add *additional details* about animal welfare and steps taken to ameliorate suffering<br>• If anesthesia, euthanasia, or any kind of animal sacrifice is part of the study, include briefly which substances and/or methods were applied<br><br>**Field Research**<br><br>Include the following details if this study involves the collection of plant, animal, or other materials from a natural setting:<br>• Field permit number<br>• Name of the institution or relevant body that granted permission	
**Data Availability**<br><br>Authors are required to make all data underlying the findings described fully available, without restriction, and from the time of publication. PLOS allows rare exceptions to address legal and ethical concerns. See the PLOS Data Policy and FAQ for detailed information.	Yes - all data are fully available without restriction

A Data Availability Statement describing where the data can be found is required at submission. Your answers to this question constitute the Data Availability Statement and **will be published in the article**, if accepted.<br><br>**Important:** Stating 'data available on request from the author' is not sufficient. If your data are only available upon request, select 'No' for the first question and explain your exceptional situation in the text box.<br><br>Do the authors confirm that all data underlying the findings described in their manuscript are fully available without restriction?	
**Describe where the data may be found in full sentences. If you are copying our sample text, replace any instances of XXX with the appropriate details.**<br><br>• If the data are **held or will be held in a public repository**, include URLs, accession numbers or DOIs. If this information will only be available after acceptance, indicate this by ticking the box below. For example: *All XXX files are available from the XXX database (accession number(s) XXX, XXX.).*<br>• If the data are all contained **within the manuscript and/or Supporting Information files**, enter the following: *All relevant data are within the manuscript and its Supporting Information files.*<br>• If neither of these applies but you are able to provide **details of access elsewhere**, with or without limitations, please do so. For example:<br><br>*Data cannot be shared publicly because of [XXX]. Data are available from the XXX Institutional Data Access / Ethics Committee (contact via XXX) for researchers who meet the criteria for access to confidential data.*<br><br>*The data underlying the results presented in the study are available from (include the name of the third party*	If the data are all contained within the manuscript and/or Supporting Information files, enter the following: All relevant data are within the manuscript and its Supporting Information files.

Powered by Editorial Manager® and ProduXion Manager® from Aries Systems Corporation

and contact information or URL).<br>• This text is appropriate if the data are owned by a third party and authors do not have permission to share the data.<br><br>* typeset	
Additional data availability information:	

Manuscript Click here to access/download;Manuscript;manuscript.docx

Title page

- **The type of manuscript:** Original article.
- **Title:** Eating Habits of Students of Health Colleges and Non-Health Colleges at the Northern Border University in the Kingdom of Saudi Arabia.
- **Running title:** Eating habits of university students at NBU.
- **Authors:**

1- **Hanaa E. Bayomy** *(the corresponding author):* Department of Public Health and Community Medicine, Faculty of Medicine, Benha University, Egypt and Department of Family and Community Medicine, Faculty of Medicine, Northern Border University, Saudi Arabia.

2- **Shmoukh Mushref Alruwaili:** Faculty of Medicine, Northern Border University, Saudi Arabia. shmoukhalrawily@gmail.com

3- **Razan Ibrahim Alsayer:** Faculty of Medicine, Northern Border University, Saudi Arabia. Razan1alsayer@gmail.com

4- **Nuof Khalid Alanazi:** Faculty of Medicine, Northern Border University, Saudi Arabia. dr.NuofAL3nazi@gmail.com

5- **Dana Ahmed Albalawi:** Faculty of Medicine, Northern Border University, Saudi Arabia. dana.alaradi2000@gmail.com

6- **Khulud Hamed Al Shammari:** Faculty of Medicine, Northern Border University, Saudi Arabia. Khuludd.212@gmail.com

7- **Mariam Moussa:** Faculty of Medicine, Benha University, Egypt. maryam20995@fmed.bu.edu.eg

Corresponding author:
Dr. Hanaa El-Sayed Bayomy, Ph.D, MD, MSc. ORCID: 0000-0001-7273-7931
Department of Public Health & Community Medicine, Faculty of Medicine, Benha University, Egypt, and the Department of Family & Community Medicine, Faculty of Medicine, Northern Border University, KSA.
Tel.: +966 59 048 9691
E mail: hana.bayuomi@fmed.bu.edu.eg; Hanaa.Sayed@nbu.edu.sa

Word count (text excluding the references, tables and abstract): 3,982.

Number of tables: 5

Number of figures: 2

Number of references: 54

Eating Habits of Students of Health Colleges and Non-Health Colleges at the Northern Border University in the Kingdom of Saudi Arabia

Abstract:

Background: Eating habits are important determinants for good health. However, young adults who have recently transitioned to university life experience stress and lack of time, which are major risk factors for poor eating habits. This study aimed to determine and compare eating patterns between students at health and non-health colleges at Northern Border University (NBU), Saudi Arabia and to identify the relationship between eating patterns and sociodemographic and psychological characteristics of students.

Methods: This comparative cross-section study was conducted on 480 students equally from health and non-health colleges at NBU from March to April 2023. A pre-designed electronic questionnaire was used to collect data on students' sociodemographic characteristics, eating habits, and diet-related psychological factors. Statistical analyses were carried out using STATA/SE version 11.2 for Windows.

Results: Unhealthy eating habits were reported by 44.6% and 41.3% of students at health and non-health colleges, respectively. Most students had irregular meals, skipped breakfast, ate fast food frequently, and consumed insufficient amounts of vegetables, fruits, and water. Parental separation, living away from family predisposed to unhealthy eating habits. Being 21-23 years old, playing sports, and high body mass index were linked to healthy eating habits. Psychological factors such as overeating until the stomach hurts and eating to feel happy were associated with unhealthy eating habits.

Conclusion: Unhealthy eating habits were prevalent among students at NBU irrespective of the type of study. Thus, implementing initiatives to promote nutrition and healthy eating habits within the university environment is crucial for health promotion and well-being among students.

Keywords: Eating habits; University students; Health colleges; Non-health colleges; Psychological factors; Northern Saudi Arabia.

Introduction :

A healthy diet is one that provides enough micronutrients and hydration to meet the body's physiologic needs. While also supporting the energetic and physiologic needs of the body without overindulging in macronutrients. For example, macronutrients (i.e., carbohydrates, proteins, and fats) provide the energy for cellular processes needed for daily functioning, and micronutrients (i.e., vitamins and minerals), on the other hand, are required in relatively small amounts for normal growth, development, metabolism, and physiologic functioning.[1]

Healthy eating also includes consistent food consumption behaviors and patterns, which is very advantageous for supporting and maintaining both physical and psychological health.[2] Furthermore, The World Health Organization places (WHO) a high priority on nutrition and maintaining a healthy diet.[3] Eating habits are the foundation of health as 25-30 percent of our well-being is determined by our eating habits.[4]

In addition, the WHO has stated that health-risk behaviors that begin in adolescence (such as unhealthy eating habits) are a major cause of disease burden in adults.[5] For example, most of the Saudi population has higher levels of consumption of refined foods and animal products than fruits and vegetables.[6] Diet-related diseases including cardiovascular disease, cancer, and stroke are always among the top three causes of death.[7] Moreover, obesity is a consequence of the interaction of poor eating habits, other environmental factors, and genetic predisposition, and plays a fundamental role in the development of coronary heart disease, hypertension, and diabetes mellitus.[8]

Many psychological factors affect eating behaviors and food choices among university students such as stress, depression, boredom, and anxiety.[9] It has been demonstrated that young adults are continually challenged to make healthy food choices after the transition from adolescence to young adulthood when independence increases.[10,11] However, young adults who have recently made the transition into university life are exposed to stress and a lack of time, which are significant public health causes for poor dietary intake.[5] Medical students have sufficient knowledge about healthy diets and thus are supposed to practice healthier eating habits than non-medical students. However, they rarely apply this knowledge.[12] Additionally, due to the dedication to their studies and clinical rotations in their respective wards, the majority of medical students do not exercise or consume healthier foods due to a shortage of time. Another reason is the continuous stress of being a medical student and the burden of their studies would have a negative impact on

their diet.[13,14] However, Medical students must develop healthy eating habits because they will be future healthcare providers. Students and physicians who decide not to live a healthy lifestyle are more reasonably to fail to promote health for their patients in the future.[13,15]

A Few studies have been conducted on the eating habits of medical and non-medical students, but in our study, we engage students from all the health colleges to compare with their colleagues from other colleges. This study aimed to increase nutritional awareness among NBU students and to spread proper nutritional practices in order to enjoy a healthy life. The specific objectives of the study were to identify and compare eating patterns between students at health colleges and non-health colleges at NBU, and to determine the relationship between students' eating patterns and their sociodemographic, lifestyle, and psychological factors.

Methods:

Study design: A comparative cross-section study.

Setting: This study was conducted on students at NBU from the first of March to the end of April 2023. NBU is located in the Northern Saudi Arabia and comprises 13 colleges located in three cities namely Arar, Rafha, and Turaif. These colleges were divided into four health colleges (College of Medicine, College of Medical Sciences, College of Pharmacy, and College of Nursing) and nine non-health colleges (College of Sciences, College of Sciences and Arts in Rafha, College of Engineering, College of Sciences and Arts in Turaif, College of Computing and Information Technology, College of Education and Arts, College of Family and Consumer Sciences, College of Business Administration, and Applied College in Arar).

Study population and sampling strategy: The estimated sample size to compare proportions between two samples was calculated using STATA/SE version 11.2 for Windows (STATA Corporation, College Station, Texas). Two samples of 240 from health colleges and non-health colleges were selected, assuming a 50% prevalence of the outcome, a 15% difference between health and non-health colleges, a 95% confidence interval, a 5% margin of error, and a power of 90%.

A convenient sample was selected from health colleges and non-health colleges at NBU. All students at NBU were candidates for the study, including both male and female students. Students with chronic conditions such as diabetes mellitus, depression, or eating disorders, those

taking long-term medications that affect body weight such as estrogen or corticosteroids, those who underwent bariatric surgery, pregnant, or athletes were excluded from the study.

Data collection:

A self-administered online questionnaire using Google Forms was used to collect data. The questionnaire contains an introductory paragraph that informs participants of the study's aims, the confidentiality of their responses, and the freedom to accept or decline participation in the study.

A predesigned and validated questionnaire that was employed in previous studies in Saudi Arabia was used to collect data on sociodemographic characteristics, eating habits, and eating-related psychological factors.[16-18] The questionnaire comprised three parts: First: Sociodemographic characteristics of the study participants including gender, age, nationality, college, academic years, marital status, tobacco smoking, physical activity, place of residence, living arrangement, household income, number of family members, parents' education, whether parents were separated or not, and whether students' mothers were working or not. Students were asked about their body weight and height to calculate the Body Mass Index (BMI)= weight in kilogram (kg)/ squared height in meters (m^2). Second: Eating habits questionnaire that inquired data on having regular meals and ten items regarding the frequency of consumption of different types of food including daily breakfast, number of meals per day, frequency of having snacks per week, frequency of vegetables and fruits consumption per week, weekly consumption of fried food and fast food, frequency of eating with family/friends, amount of water intake pe[illegible]ay, and the type of food consumed. Correct responses to these items were coded as "1" and wrong responses were coded as "0". The total dietary score equals the sum of all responses and ranges from zero to 10.[14] Third: Six eating-related psychological factors.[19,20] These include eating due to loneliness, being upset or nervous, feeling bored, feeling happy, loss of control when it comes to food, and eating too much until the stomach hurts. Responses to these items were either "rarely", "sometimes', or "often".

Questions about chronic illnesses, medications taken, prior bariatric surgery, pregnancy, and being athletes were included to exclude non-illegible students.

Ethical consideration: This study was approved by the Local Committee of Bioethics (HAP-09-A-043) at NBU no. (27/44/H). Informed consent was obtained from all students prior to their participation.

Data analysis: The collected data were entered, presented, and analyzed using the computerized statistical package STATA/SE version 11.2 for Windows (STATA Corporation, College Station, Texas), and MS Excel. Data were summarized using mean ± Standard Deviation (SD), minimum and maximum values for numerical data, and frequency and percentage for qualitative data. Categorical data were compared using the Chi-square test (x^2) and the Fisher Exact Test (FET) as appropriate. The Mann-Whiteny test (z) was used to compare numerical data that was found not normally distributed using the Shapiro-Wilk W test. The median dietary score was used to dichotomize dietary scores and logistic regression of adequate dietary habits (score≥4) conditioned on potential sociodemographic and psychological factors was carried out to identify significant predictors. Statistical significance was considered at P<0.05.

Results:

The study recruited 480 eligible Saudi students equally from health and non-health colleges. Table 1 shows sociodemographic characteristics for the studied students. Regarding the students from health colleges, 63.7% were females, with an average age of 21.3(±1.8). In the non-health colleges, most of the participants were females 69.6% and their mean age was 21.0(±1.7). More than half of the students from health colleges were from clinical years (fourth academic year - internship) while in the non-health colleges, 63.75% of students were from first to third academic years (P<0.001). Most of the participants were single, living in owned homes with their families, had household monthly income between 3000-10000 SR. More than half of the participants had 6-9 family members and less than 10% had separated parents and were smokers.

Students at health colleges were more likely to have highly educated parents (P=0.001) and working mothers (P<0.001) than those of non-health colleges. No regular physical activity was reported by 45.8% and 40.4% of students of health and non-health colleges, respectively (P=0.03). There were no significant differences in BMI between students of health and non-health colleges.

Figure 1 shows the frequency distribution of eating habits reported by the study participants. Only 37.1% and 39.2% of students from health and non-health colleges, respectively,

reported having regular meals. Less than half of the participants ate breakfast daily and received three or more daily meals with three or more snacks per week. Most students ate vegetables and legumes less than three times a week (60.4% and 62.9% for students of health and non-health colleges, respectively) as well as fruits (85.0% and 83.7% for students of health and non-health colleges, respectively). Around 57.1% of students from health colleges and 63.3% of students from non-health colleges students ate fried foods less than three times a week. Only 5% of participants reported that they rarely eat fast food. About 50.4% and 47.1% of students at health and non-health colleges, respectively ate with their family and friends daily. Most of the students reported having less than two liters of water per day (71.7% and 73.7% for students of health and non-health colleges, respectively). More than half of students (54.2% and 59.2% for students of health and non-health colleges, respectively) ate a variety of food in balance. There were no significant differences in eating habits between student of health and non-health colleges.

Psychological factors affecting eating habits among studied students are demonstrated in Figure 2. More than half of the students of health and non-health colleges reported that they rarely ate because they felt lonely (52.9% and 51.2%), felt completely out of control about food (59.6% and 55.4%), ate so much that their stomach hurts (58.3% and 52.1%), and ate because of feeling upset (53.7% and 51.7%). While 49.2% and 45.8% of students from health and non-health colleges, respectively, reported that they sometimes ate because they felt bored and 48.7% and 47.9%, respectively, sometimes ate when they felt happy. These differences were statistically non-significant.

A total of 133 students (55.4%) from health colleges had adequate eating habits (Table 2). These were more likely in students aged 21-23 years (P=0.02) and those living with their families (P=0.04). Parental separation and physical inactivity were associated with inadequate eating habits at P=0.02 and P=0.047, respectively. More than half of students (55.6%) with adequate eating habits were of normal weight (P=0.03).

Table 3 shows that a total of 141 students (58.7%) from non-health colleges had adequate eating habits. Adequate eating habits were less likely among students with separated parents (P=0.01) and those who practice sports (P=0.001).

Table 4 shows the relationship between psychological factors and eating habits among students of health and non-health colleges. For students from non-health colleges, adequate eating habits were more frequent in students who rarely felt completely out of control about food (P=0.007) and ate so much that their stomach hurts (P<0.001). About 56% of students who rarely ate because of feeling upset or nervous had adequate eating habits (P=0.08). Psychological factors had non-significant differences in eating habits among students at health colleges.

A logistic regression model was constructed to determine the Odds Ratio and 95% Confidence Intervale (OR and 95% CI) for adequate eating habits conditioned on significant predictors (Table 5). For students of health colleges, adequate eating habits were more likely among elder students aged 21-23 years (OR=2.58 and 95% CI=1.33-4.99; P=0.005), and those who lived with their families (OR=3.04 and 95%CI= 1.10-8.41; P=0.03). While students with separated parents were less likely to have healthy eating habits (OR=0.23 and 95%CI= 0.07-0.75; P=0.01). Regular physical activity was associated with appropriate eating habits (P<0.05). Normal-weight students were more likely to eat properly than underweight students (OR=2.87 and 95%CI= 1.23-6.69; P=0.01).

For students at non-health colleges, adequate eating habits were less likely among students with separated parents (OR=0.19 and 95%CI= 0.06-0.60; P=0.005) and those who reported that they sometimes eat because of feeling happy (OR=0.40 and 95%CI= 0.18-0.87; P=0.02). Exercising regularly for more than two days per week was associated with an increased likelihood of healthy eating (OR=3.61 and 95%CI= 1.81-7.20; P<0.001). Students who rarely or sometimes ate so much until their stomach hurts were more likely to eat properly compared to those who often did this (OR=9.19 and 95%CI= 3.21-26.37; P<0.001 and OR=4.73 and 95%CI= 1.63-13.69; P=0.004, respectively).

Discussion:

This study revealed that 44.6% and 41.3% of students at health and non-health colleges, respectively, had inadequate eating habits, with an average eating habit score of 3.9. Most students had irregular meals, skipped breakfast, and consumed insufficient vegetables, fruits, and water. In addition, a considerable proportion of students frequently consumed fried and fast food. Similar results were reported among university students in Saudi Arabia. Most medical students at King Abdulaziz University, Jeddah had unhealthy dietary habits including irregular meals, inadequate

intake of fruits and vegetables, and frequent eating of fried and fast foods with an average eating habit score of 4.2.[18] Again, the majority of medical students from five Saudi universities had inadequate eating habits.[16] Moreover, studies in Saudi Arabia to assess eating habits in university students using the Eating Attitude Test (EAT-26) revealed prevalence of high-risk dietary disorders ranging from 29.4% to 35.4%.[21-23] Also, a study in the United Arab Emirates [24] showed poor eating habits among university students such as not meeting the recommended intake of vegetables, fruits, and water.[20] Outside the Gulf region, poor eating habits were reported by most university students in Malaysia [14] and Darussalam [25]. While 22.75% of medical students in Pakistan [26] and 17% in Lebanon [27] were at high risk of eating disorders. The differences in eating habits can be due to different cultural and environmental factors besides the variable numbers of students involved.

Obesity has been linked to poor dietary habits .[8] Our results showed no differences in BMI between students of health and non-health colleges with 35.4% and 29.7% of students from health and non-health colleges were overweight/obese, respectively. The prevalence rates of overweight/obesity in recent studies on Saudi university students were 31% [21] and 34.7%.[28] This was comparable to findings in the UAE.[20,24] The prevalence of overweight/obesity in university students in other Arab countries were 36% in Jordan [29] and 35.9% in Upper Egypt.[30] While smaller proportion of university students were overweight/obese in Iran (21.3%) [31] and Malaysia (24.44%).[32]

In the present study, only 27.1% and 35.8% of students at health and non-health colleges practiced sports more than two days per week while 45.8% and 40.4% of students, respectively from health and non-health colleges reported not playing sports. These findings were consistent in Saudi Arabia.[18,33] Al-Nakeeb et al reported lower level of physical activity among young Saudi people (26%) compared to adolescents in two British cities (72.3-77.2%).[33] In the UAE, 32.7-39% of university students did not practice sports.[20,34] The prevalence of physical activity in the countries of the Gulf Cooperation Council (GCC) is generally low with men more active than women due to socio-cultural and environmental factors of the Gulf region, which predispose to many non-communicable diseases including obesity.[35]

Our study revealed that more than one third of students ate regular meals, which was comparable to previous studies in Qassim (36.7%) [36] and in Abha (31%) [17] while was lower than that reported in Jeddah (50.5%).[18] Studies outside Saudi Arabia reported higher prevalence of

regular meals in university students such as in Malaysia (57.6%) [14], Lebanon (61.4%) [37], Darussalam (74.6%) [25], and China (83.6%).[38]

In this study, 40% and 45% of students at the health and non-health colleges ate breakfast daily. Comparable results were reported among university students in the UAE (45.4%) [20], Darussalam (42.6%) [25], and Malaysia (43.9%).[14] While 41% and 68.7% of anemic and non-anemic students in Bangladesh had breakfast daily.[39] However, smaller proportions were reported among Saudi medical students (34.7%) [18] and university students in Lebanon (31.8%).[37] While higher proportion (59.7%) of university students in coasta[illegible]enya had daily breakfast.[40] Skipping breakfast can be due to lack of time, reduced appetite and not feeling hungry early morning, and can negatively affect the academic performance of students and their other eating habits e.g., frequent snacks. [41,42]

About half of the studied students had snacks three or more times per week. Consumption of fried food was reported by 42.9% and 36.7% of students of health and non-health colleges at least three times per week, and only 5% rarely ate fast food. While 60.4% and 47.3% of Saudi medical students in Jeddah reported having snacks and eating fried foods at least three times a week, respectively, and 6.1% never eating fast food.[18] In Abha, 35.96% of university male students reported eating snacks daily.[17] In addition, 88.7% of Saudi male students reported eating fast food at least one time per week.[43] Meanwhile, 42.4% of Malaysian students had snacks three or more times per week.[14] While 82.2% of students at Universiti Brunei Darussalam reported eating snacks between meals and 60.7% ate fried food 3-5 times per week.[25] In Kenya, 47.2% of university students reported eating fried food 3 or more times per week and only 15.3% ate with snacks on daily basis.[40]

The WHO recommended daily consumption of five servings of fruits and vegetables that is equivalent to 400 grams per day.[44] Also, the Saudi Ministry of Health recommended consumption of 3-5 servings of vegetables and 2-4 servings of fruits per day for healthy food and protection against many diseases.[45] However, most students in the present study reported eating vegetables and fruits less than three times a week. Previous studies in Saudi Arabia reported similar results.[17,18,46] Correspondingly, only 28.7% and 34% of university students in the UAE ate fruits and vegetables daily [20] and 30.5% of Lebanese university students ate colored vegetables daily.[37] Meanwhile 42.9% and 41.9% of university students in Brunei ate vegetables and fruits 3-5 times

per week [25] and most Kenyan students reported eating vegetables (87.5%) and fruits (69.4%) daily.[40]

In this study, most students drank less than the recommended daily water intake (two liters and two and half liters for females and males, respectively according to the European Food Safety Authority).[47] In agreement, 90% of Saudi medical students reported drinking less than two liters of water per day.[18] Similarly, more than four fifths of UAE university students consumed less than two liters per day.[20] In Iran, the average amount of daily fluid intake by university students was 1.7 liters.[48] Outside the Gulf area, 57.8% of university students reported drinking at least two liters of water per day.[25]

About half of the studied students reported eating with their families and friends daily and more than half ate a variety of food in balance. These results agreed with previous studies in Saudi Arabia.[17,18] However, a smaller proportion of students reported eating with their families and friends in Lebanon (42.7%).[37] Meanwhile most Malaysian students (81.8%) ate with their families.[14]

This study revealed that most students rarely ate because of feeling lonely or nervous, they rarely felt completely out of control about food, and rarely ate so much that their stomachs hurt. While about one fifth reported they often ate because of boredom or happiness. These findings agree with previous records of Saudi medical students in Jeddah where only 10-21% of students reported that they often eat due to psychological factors.[18] In the UAE, more than 80% of the University of Sharjah students ate out of boredom or happiness, and more than half ate out of sadness.[20] The association between psychological factors and eating habits has been proved. Moynihan et al reported that boredom increased caloric consumption to distract from this feeling.[49] In addition, food intake was significantly increased in the positive mood condition compared to the neutral condition while the negative condition had no effect on food consumption. Moreover, there was a positive correlation between the total calories consumed and improved mood.[50] A meta-analysis revealed that induced positive and negative mood conditions were associated with increased eating in both healthy and patients with eating disorders.[51] Mood changes associated with changes in food choices and quantities. The link between mood and food can be mediated by hormones such as ghrelin and leptin and neurotransmitters such as dopamine and serotonin.[52]

In this study, 55.4% and 58.75% of students of health and non-health colleges reported sound eating habits. Healthy eating was more likely in students at health colleges who had normal body weight, aged 21-23 years, and those living with their families. While parental separation and physical inactivity were associated with inadequate eating habits among students of both health and non-health colleges. Similarly, higher dietary scores were more likely among Saudi medical students who lived with their families, whose parents were not separated, were physically active, never smokers, and lived in an owned place (P<0.05).[18] In line with these findings, unhealthy feeding was more frequent in unmarried students, those not living with their families, smokers, and physically inactive [20]. It was suggested that lack of family supervision in students living alone predisposed to unhealthy habits such as unhealthy food choices and lack of physical activity.[53]

In the present study, psychological factors such as feeling out of control about food, too much eating till stomach hurts, and eating because of being upset or nervous were unlikely among students with adequate eating habits. Similarly, Alzahrani et al recorded higher eating scores among medical students who never ate out of feeling lonely, felt out of control when it came to food, ate until their stomach hurt, ate because of feeling upset or nervous, or ate because of boredom compared to those who did so often or sometimes (P<0.05).[18] In Kuwait, stress was associated with consumption of unhealthy food such as fast-food rich in fats, sugars, and soft drinks particularly in female students.[54] In agreement, unhealthy dietary habits and obesity were associated with uncontrollable eating.[20,53]

This comparative cross-sectional study examined dietary habits among students of both health and non-health colleges at the NBU. A wide range of underlying factors for unsatisfactory dietary habits have been studied including sociodemographic characteristics, lifestyle, and psychological factors. The study presented important factors with potential implications for improving the dietary habits of university students, which could have long-term effects on their health and well-being.

However, our results are based on self-reported data with a risk of bias due to social desirability, over-reporting of healthy habits, or under-reporting of unhealthy habits. The absence of a specified period of recall and the inability to establish causality are also important considerations. Additionally, the fact that factors like chronic diseases and dietary disorders have

not been studied highlights a potential area for future research. Conducting larger-scale studies across diverse population subgroups would indeed provide a more comprehensive understanding of eating habits and the underlying factors influencing them.

Finally, unhealthy eating habits were prevalent among students at NBU irrespective of the type of study. This was evident in terms of irregular meals, skipping breakfast, and insufficient intake of fruits, vegetables, and water. Young age, parental separation, lack of physical activity, living away from family, and low BMI were associated with unhealthy eating habits. Some psychological factors, such as overeating until the stomach hurts and eating to feel happy, increase the risk of eating unhealthy food. Given these findings, implementing initiatives to promote nutrition and healthy eating habits within the university environment is crucial. This could involve educational campaigns focused on nutrition, as well as integrating nutritional education into the university curricula. Encouraging and supporting students in adopting healthier diets and lifestyles can have long-lasting benefits for their overall well-being and academic performance.

Availability of data and materials: The data that support the findings of this study are available from the corresponding author upon request.

Competing interests: The authors declare that they have no competing interests.

Funding: The authors declare that no funds, grants, or other support were received during the preparation of this manuscript.

Author’s contributions: All authors contributed to the study conception, design, and material preparation. Data collection and data entry were performed by [SMA], [RIA], [NKA], and [DAA]. Statistical analysis was carried out by [HB] and [MM]. The first draft of the manuscript was written by [HB] and [SMA], and all authors commented on previous versions of the manuscript. All authors read and approved the final manuscript.

Acknowledgment: The authors are thankful to all students who agreed to participate in this study.

References

1. Cena H, Calder PC. Defining a healthy diet: Evidence for the role of contemporary dietary patterns in health and disease. *Nutrients.* 2020 Jan 27;12(2):334.doi: 10.3390/nu12020334. PMID: 32012681; PMCID: PMC7071223.

2. Musaiger AO, Kalam F. Dietary habits and lifestyle among adolescents in Damascus, Syria. *Ann Agric Environ Med.* 2014;21(2):416-9.doi: 10.5604/1232-1966.1108616. PMID: 24959801.

3. Ngoma T. World Health Organization cancer priorities in developing countries. *Ann Oncol.* 2006;17(Suppl 8):viii9-viii14.doi: 10.1093/annonc/mdl982. PMID: 16801342.

4. Skemiene L, Ustinaviciene R, Piesine L, Radisauskas R. Peculiarities of medical students' nutrition. *Medicina (Kaunas)*. 2007;43(2):145-52.English, Lithuanian. PMID: 17329950.

5. Sogari G, Velez-Argumedo C, Gomez MI., Mora C. College students and eating habits: A study using an ecological model for healthy behavior. *Nutrients*. 2018;10(12):1823.doi: 10.3390/nu10121823. PMID: 30477101; PMCID: PMC6315356.

6. Al-Hazzaa HM, Abahussain NA, Al-Sobayel HI, Qahwaji DM, Musaiger AO. Physical activity, sedentary behaviors and dietary habits among Saudi adolescents relative to age, gender and region. *Int J Behav Nutr Phys Act*. 2011;8(140).doi: 10.1186/1479-5868-8-140. PMID: 22188825; PMCID: PMC3339333.

7. Small M, Bailey-Davis L, Morgan N, Maggs J. Changes in eating and physical activity behaviors across seven semesters of college: living on or off campus matters. *Health Educ Behav.* 2013;40(4):435-41.doi: 10.1177/1090198112467801. Epub 2012 Dec 10. PMID: 23232092; PMCID: PMC4186223.

8. Berenson GS, Bogalusa Heart Study group. Health consequences of obesity. *Pediatr Blood Cancer.* 2012;58(1):117-21.

9. Hootman KC, Guertin KA, Cassano PA. Stress and psychological constructs related to eating behavior are associated with anthropometry and body composition in young adults. Appetite

2018;125:287-94.doi: 10.1016/j.appet.2018.01.003. Epub 2018 Jan 5. PMID: 29309851; PMCID: PMC5878735.

10. LaCaille LJ, Dauner KN, Krambeer RJ, Pedersen J. Psychosocial and environmental determinants of eating behaviors, physical activity, and weight change among college students: a qualitative analysis. *J Am Coll Health.* 2011;59(6):531-8.doi: 10.1080/07448481.2010.523855. PMID: 21660808.

11. Stok FM, Renner B, Clarys P, Lien N, Lakerveld J, Deliens T. Understanding eating behavior during the transition from adolescence to young adulthood: a literature review and perspective on future research directions. *Nutrients*. 2018;10(6):667.doi: 10.3390/nu10060667. PMID: 29794986; PMCID: PMC6024552.

12. Sajwani RA, Shoukat S, Raza R, Shiekh MM, Rashid Q, Siddique MS, et al. Knowledge and practice of healthy lifestyle and dietary habits in medical and non-medical students of Karachi, Pakistan. J Pak Med Assoc 2009;59(9):650-5.PMID: 19750870.

13. Agha SA, Agha MA, Usman G, Agha Z. Assessment of the perceptions of health among medical students. *Gomal Journal of Medical Sciences.* 2011;9(2).

14. Ganasegeran K, Al-Dubai S, Qureshi A, Al-abed A, Abdul Manaf MR, Aljunid S. Social and psychological factors affecting eating habits among university students in a Malaysian medical school: A cross-sectional study. *Nutr J.* 2012 Jul 18;11(1):48.doi: 10.1186/1475-2891-11-48. PMID: 22809556; PMCID: PMC3418187.

15. Helal HEB, El-Awady MA. Egyptian Physicians' Lifestyle Behaviors and Health Promotion Activities Towards Patients with Chronic Non-Communicable Diseases. *Medical Science.* 2022;26(121): ms102e2071.doi: https://doi.org/10.54905/disssi/v26i121/ms102e2071.

16. Bantan AK, Etarji SMM, Mohamed DS, Elkersh JMD, Alharbi TS, Khan ZAM. Eating habits among medical students, Jeddah, Saudi Arabia. *IJMDC.* 2021;5(5):1119-1126.doi:https://doi.org/10.24911/IJMDC.51-1611165681 .

17. Alshahrani M, Chandramoha S. A cross sectional study on prevalence of obesity and its association with dietary habits among college students in Abha, Saudi Arabia. *International*

Journal of Community Medicine and Public Health. 2017 Mar 1;4(5):1406-1412.doi:https://doi.org/10.18203/2394-6040.ijcmph20171747.

18. Alzahrani SH, Saeedi AA, Baamer MK, Shalabi AF, Alzahrani AM. Eating Habits Among Medical Students at King Abdulaziz University, Jeddah, Saudi Arabia. *Int J Gen Med.* 2020 Mar 5;13(null):77-88.doi: 10.2147/IJGM.S246296.

19. Kagan DM, Squires RL. Compulsive eating, dieting, stress, and hostility among college students. *Journal of College Students personnel.* 1984;25(3):213-20.

20. Cheikh Ismail L, Osaili TM, Mohamad MN, Hashim M, Stojanovska L, Al Daour R, et al. Psychosocial factors affecting dietary habits of university students: A cross-sectional study. *Heliyon*. 2022;8(6):e09768.doi: 10.1016/j.heliyon.2022.e09768. PMID: 35789869; PMCID: PMC9249847.

21. Ghamri RA, Alahmari AM, Alghamdi LS, Alamoudi SF, Barashid MM. Prevalence and predictors of eating disorders: A cross-sectional survey of medical students at King Abdul-Aziz University, Jeddah. *Pak J Med Sci.* 2022;38(6):1633-8.doi: 10.12669/pjms.38.6.5033. PMID: 35991250; PMCID: PMC9378390.

22. Abd El-Azeem Taha AA, Abu-Zaid HA, El-Sayed Desouky D. Eating Disorders Among Female Students of Taif University, Saudi Arabia. *Arch Iran Med.* 2018;21(3):111-7.PMID: 29688736.

23. Aisha MA, Salwa AA, Moataza MAW, Leila AB, Bader AA. Eating disorders and associated risk factors among Imam Abdulrahman bin Faisal university preparatory year female students in Kingdom of Saudi Arabia. *Saudi Med J.* 2018 Sep 1;39(9):910-21.doi: 10.15537/smj.2018.9.23314. PMID: 30251735; PMCID: PMC6201000.

24. Radwan H, Hasan HA, Najm L, Zaurub S, Jami F, Javadi F, et al. Eating disorders and body image concerns as influenced by family and media among university students in Sharjah, UAE. *Asia Pac J Clin Nutr.* 2018;27(3):695-700.doi: 10.6133/apjcn.062017.10. PMID: 29737819.

25. Yun TC, Ahmad SR, Quee DKS. Dietary habits and lifestyle practices among university students in Universiti Brunei Darussalam. Malays J Med Sci. 2018;25(3):56-66.doi: 10.21315/mjms2018.25.3.6. Epub 2018 Jun 28. PMID: 30899187; PMCID: PMC6422551.

26. Memon AA, Adil SE-R, Siddiqui EU, Naeem SS, Ali SA, Mehmood K. Eating disorders in medical students of Karachi, Pakistan-a cross-sectional study. *BMC Res Notes.* 2012;5(84).doi: 10.1186/1756-0500-5-84. PMID: 22296613; PMCID: PMC3395848.

27. Bizri M, Geagea L, Kobeissy F, Talih F. Prevalence of Eating Disorders Among Medical Students in a Lebanese Medical School: A Cross-Sectional Study. *Neuropsychiatr Dis Treat.* 2020 Aug 3;16:1879-87.doi: 10.2147/NDT.S266241. PMID: 32801721; PMCID: PMC7414930.

28. Makkawy E, Alrakha AM, Al-Mubarak AF, Alotaibi HT, Alotaibi NT, Alasmari AA, et al. Prevalence of overweight and obesity and their associated factors among health sciences college students, Saudi Arabia. *J Family Med Prim Care.* 2021;10(2):961-7.doi: 10.4103/jfmpc.jfmpc_1749_20. Epub 2021 Feb 27. PMID: 34041105; PMCID: PMC8138400.

29. Al-Awwad NJ, Al-Sayyed HF, Zeinah ZA, Tayyem RF. Dietary and lifestyle habits among university students at different academic years. *Clin Nutr ESPEN.* 2021;44:236-42.doi: 10.1016/j.clnesp.2021.06.010. Epub 2021 Jun 26. PMID: 34330472.

30. Hafez SM, Ghazawy ER, Mahfouz EM, Abd-El Rahman TA, Emam SA. Obesity/Overweight among University students. Minia, Egypt. *Minia Journal of Medical Research.* 2022;33(3):30-6.

31. Mitic N, Popovic L, Milic M, Radic I, Popovic B. Prevalence of Overweight and Obesity among Students of University in Pristina/Kosovska Mitrovica, according to Different Anthropometric Indices. *Iran J Public Health.* 2021;50(9):1919-21.doi: 10.18502/ijph.v50i9.7078. PMID: 34722393; PMCID: PMC8542813.

32. Pitil PP, Ghazali SR. Overweight and obesity: a study among university students in Sarawak, Malaysia. International Journal of Health Promotion and Education 2022;1-13.doi: 10.1080/14635240.2022.2040380.

33. Al-Nakeeb Y, Lyons M, Collins P, Al-Nuaim A, Al-Hazzaa H, Duncan MJ, et al. Obesity, physical activity and sedentary behavior amongst British and Saudi youth: a cross-cultural study. Int J Environ Res Public Health 2012 Apr;9(4):1490-506.doi: 10.3390/ijerph9041490. Epub 2012 Apr 16. PMID: 22690207; PMCID: PMC3366625.

34. Matthews JD. Health literacy among female university students in the United Arab Emirates. *International Journal of Health Promotion and Education*. 2018 Nov 2;56(6):298-308.doi: 10.1080/14635240.2018.1536524.

35. Mabry RM, Reeves MM, Eakin EG, Owen N. Evidence of physical activity participation among men and women in the countries of the Gulf Cooperation Council: a review. *Obes Rev.* 2010;11(6):457-64.doi: 10.1111/j.1467-789X.2009.00655.x. Epub 2009 Sep 29. PMID: 19793376.

36. Al-Rethaiaa AS, Fahmy AE, Al-Shwaiyat NM. Obesity and eating habits among college students in Saudi Arabia: a cross sectional study. *Nutr J.* 2010 Sep 19;9(39).doi: 10.1186/1475-2891-9-39. PMID: 20849655; PMCID: PMC2949783.

37. Yahia N, Achkar A, Abdallah A, Rizk S. Eating habits and obesity among Lebanese university students. *Nutr J.* 2008;7(32).doi: 10.1186/1475-2891-7-32. PMID: 18973661; PMCID: PMC2584644.

38. Sakamaki R, Toyama K, Amamoto R, Liu CJ, Shinfuku N. Nutritional knowledge, food habits and health attitude of Chinese university students -a cross sectional study-. *Nutr J.* 2005;4:1-5.

39. Shill KB, Karmakar P, Kibria MG, Das A, Rahman MA, Hossain MS, et al. Prevalence of iron-deficiency anaemia among university students in Noakhali region, Bangladesh. *J Health Popul Nutr.* 2014;32(1):103-10.PMID: 24847599; PMCID: PMC4089078.

40. Mogeni BK, Ouma LO. Dietary patterns, behaviours, and their associated factors among university students in coastal Kenya. *Cogent Food & Agriculture.* 2022 Dec 31;8(1):2132873.doi: 10.1080/23311932.2022.2132873.

41. Savige G, MacFarlane A, Ball K, Worsley A, Crawford D. Snacking behaviours of adolescents and their association with skipping meals. *Int J Behav Nutr Phys Act*. 2007;4(36).doi: 10.1186/1479-5868-4-36. PMID: 17868479; PMCID: PMC2045092.

42. Taha Z, Rashed AS. The effect of breakfast on academic performance among high school students in Abu Dhabi. *Arab Journal of Nutrition and Exercise*. 2017;2:40.

43. Sabra A, Taha Z, Sebiany A, Al-Kurashi N, Al-Zubier A. Coronary heart disease risk factors: Prevalence and behavior among male university students in Dammam City, Saudi Arabia. *J Egypt Public Health Assoc.* 2007 Feb 1;82(1-2):21-42.PMID: 18217323.

44. World Health Organization. Fruit and vegetables for health : report of the Joint FAO/WHO Workshop on Fruit and Vegetables for Health, 1-3 September 2004, Kobe, Japan. https://apps who int/iris/handle/10665/43143 2005 [cited 2023 Oct 23];Available from: URL: https://apps.who.int/iris/handle/10665/43143

45. Al-Dkheel Mushary. The healthy Food Palm. Dietary Guidelines for Saudis. t ly/TE4rh 2012 [cited 2023 Jan 9];Available from: URL: https://www.moh.gov.sa/en/Ministry/MediaCenter/Publications/Documents/final%20english%20%20%D8%A7%D9%84%D9%83%D8%AA%D8%A7%D8%A8%20%D8%A7%D9%84%D8%B9%D9%84%D9%85%D9%8A%20%D8%A5%D9%86%D8%AC%D9%84%D9%8A%D8%B2%D9%8A.pdf

46. AL-Otaibi H. The Pattern of Fruit and Vegetable Consumption among Saudi University Students. *Glob J Health Sci.* 2014 Feb 28;6:155-62.

47. EFSA Panel on Dietetic Products, Nutrition, and Allergies (NDA). Scientific opinion on dietary reference values for water. *EFSA Journal*. 2010;8(3):1459. https://doi.org/10.2903/j.efsa.2010.1459.

48. Balaghi S, Faramarzi E, Mahdavi R, Ghaemmaghami J. Fluids intake and beverage consumption pattern among university students. *Health Promot Perspect.* 2011;1(1):54-61.doi: 10.5681/hpp.2011.005. PMID: 24688900; PMCID: PMC3963611.

49. Moynihan AB, Tilburg WAP, Igou ER, Wisman A, Donnelly AE, Mulcaire JB. Eaten up by boredom: consuming food to escape awareness of the bored self. *Front Psychol.* 2015;6:369.doi: 10.3389/fpsyg.2015.00369. PMID: 25883579; PMCID: PMC4381486.

50. Bongers P, Jansen A, Havermans R, Roefs A, Nederkoorn C. Happy eating. The underestimated role of overeating in a positive mood. *Appetite*. 2013;67:74-80.doi: 10.1016/j.appet.2013.03.017. Epub 2013 Apr 10. PMID: 23583314.

51. Cardi V, Leppanen J, Treasure J. The effects of negative and positive mood induction on eating behaviour: A meta-analysis of laboratory studies in the healthy population and eating and weight disorders. *Neurosci Biobehav Rev.* 2015;57:299-309.doi: 10.1016/j.neubiorev.2015.08.011. Epub 2015 Aug 20. PMID: 26299807.

52. Singh M. Mood, food, and obesity. *Front Psychol*. 2014 Sep 1;5:925.

53. Moore J, Harre N. Eating and activity: the importance of family and environment. *Health Promot J Aust*. 2007 Jan 1;18(2):143-8.doi: 10.1071/he07143. PMID: 17663650.

54. Ahmed F, Al-Radhwan L, Al-Azmi GZS, Al-Beajan M. Association between stress and dietary behaviours among undergraduate students in Kuwait: gender differences. *J Nutr Health Sci*. 2014;1(1):14-6.

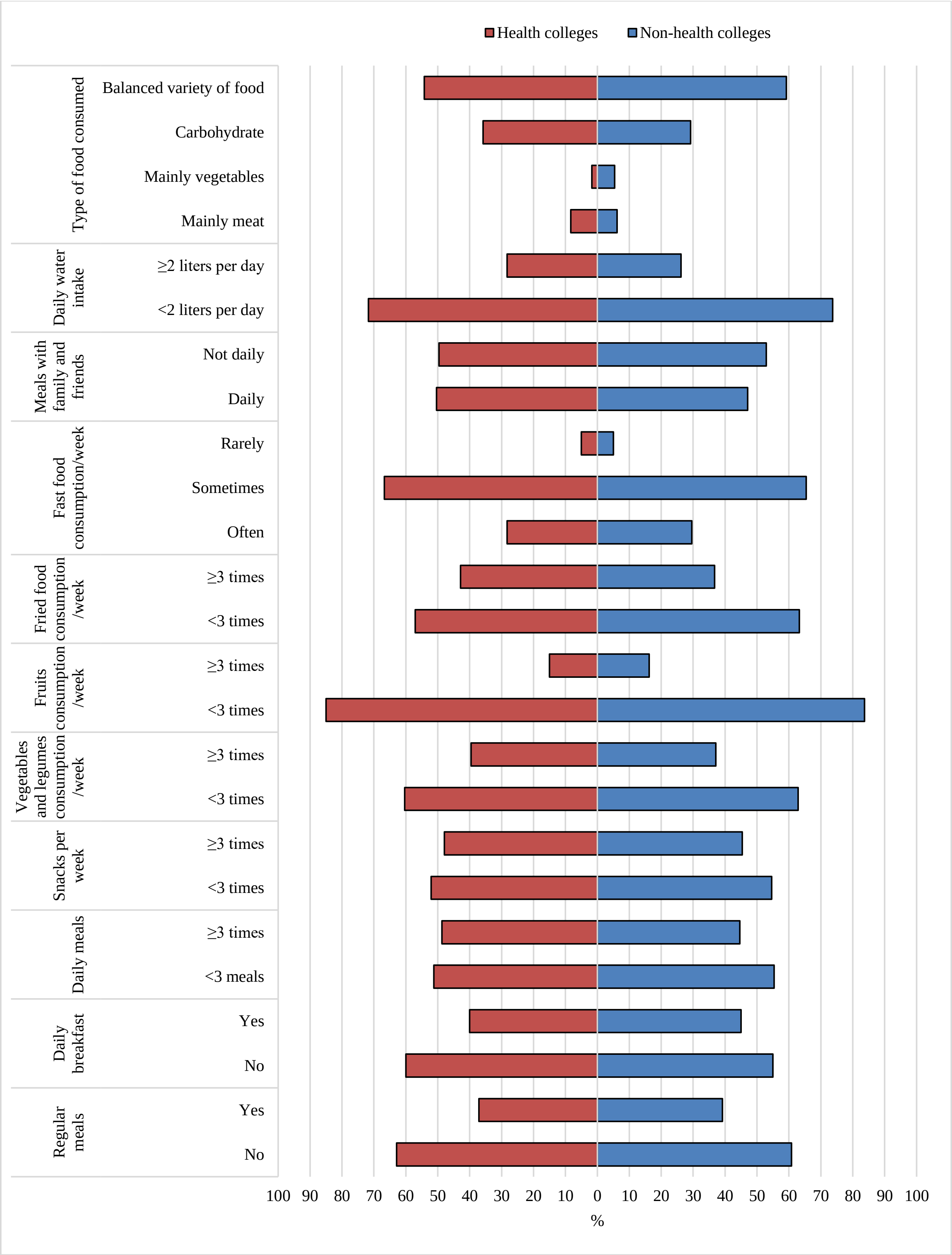

Figure 1: Eating habits among students at health and non-health colleges, Northern Border University

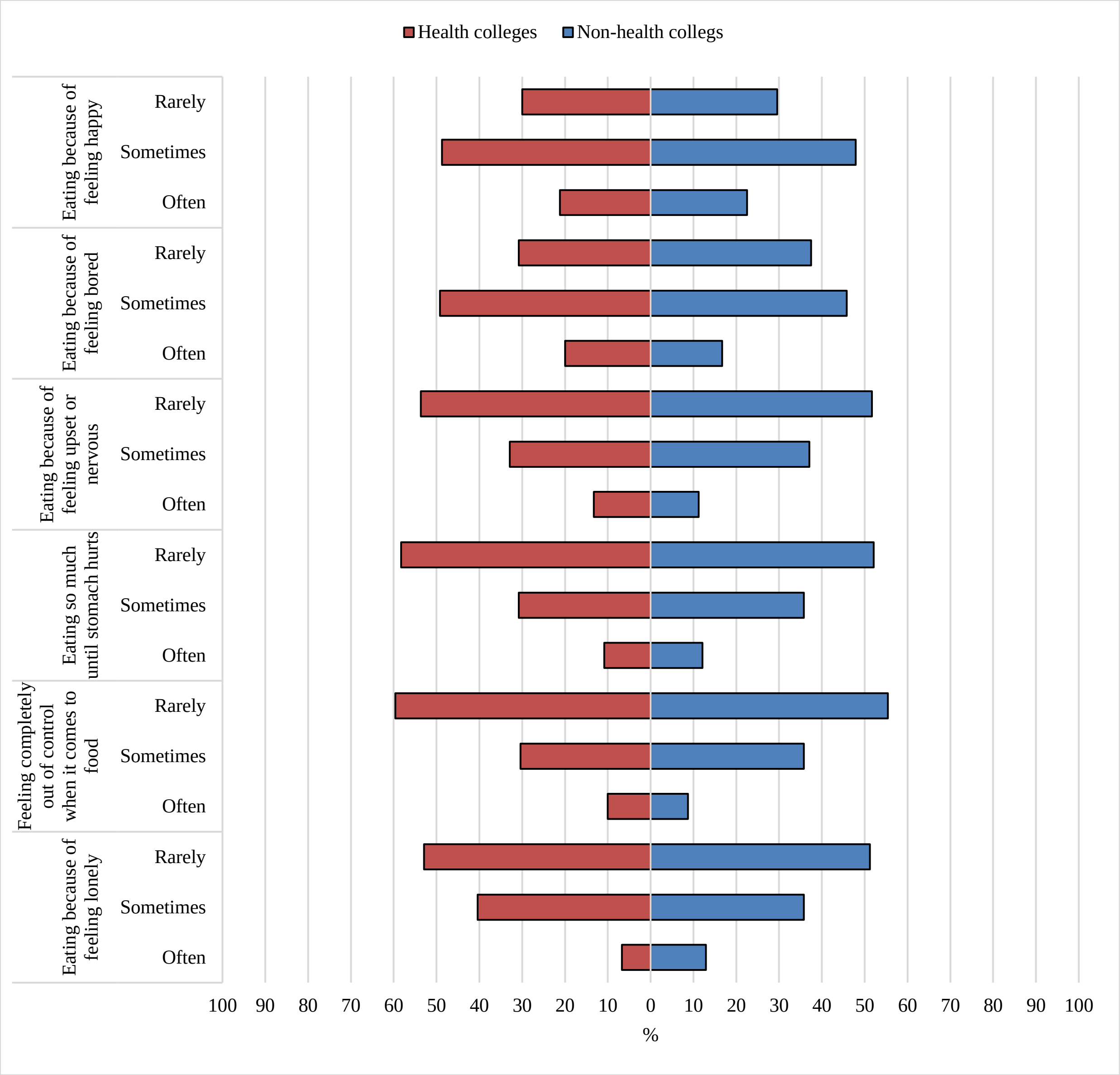

Figure 2: Psychological factors affecting eating habits among students at health and non-health colleges, Northern Border University

Table 1: Sociodemographic characteristics of students at health and non-health colleges, NBU

Characteristic		Health colleges (N.=240)		Non-health colleges (N.=240)		X^2	P
		N.	%	N.	%		
Gender	Female	153	63.7	167	69.6	1.84	0.17
	Male	87	36.2	73	30.4		
Age (year)	<21	88	36.7	108	45.0	3.49	0.17
	21-23	102	42.5	87	36.2		
	≥23	50	20.8	45	18.7		
Academic year	First	33	13.7	36	15.0	46.74	<0.001
	Second	30	12.5	57	23.7		
	Third	51	21.2	60	25.0		
	Fourth	63	26.2	70	29.2		
	Fifth	29	12.1	17	7.0		
	Sixth	15	6.2	0	0.0		
	Internship	19	7.9	0	0.0		
Marital status	Single	231	96.2	220	91.7	FET	0.06
	Married	9	3.7	18	7.5		
	Divorced	0	0.0	2	0.8		
Place of residence	Owned	205	85.4	197	82.1	0.98	0.32
	Rented	35	14.6	43	17.9		
Living arrangement	Live alone	23	9.6	12	5.0	3.73	0.05
	Live with family	217	90.4	228	95.0		
Household monthly income (SR)	3,000-10,000	207	86.2	213	88.7	0.68	0.41
	>10,000	33	13.7	27	11.2		
Number of family members	≤5	38	15.8	35	14.6	0.16	0.92
	6-9	138	57.5	139	57.9		
	≥10	64	26.7	66	27.5		
Mothers' educational level	Not educated	18	7.5	20	8.3	16.14	0.001
	Low	30	12.5	45	18.7		
	Moderate	50	20.8	76	31.7		
	High	142	59.2	99	41.2		
Fathers' educational level	Not educated	11	4.6	21	8.7	16.35	0.001
	Low	20	8.3	24	10.0		
	Moderate	72	30.0	101	42.1		
	High	137	57.1	94	39.2		
Separated parents	No	223	92.9	221	92.1	0.12	0.73
	Yes	17	7.1	19	7.9		
Working mother	No	117	48.7	168	70.0	22.46	<0.001
	Yes	123	51.2	72	30.0		
Smoking	No	222	92.5	220	91.7	0.11	0.73
	Yes	18	7.5	20	8.3		
Regular physical activity	No	110	45.8	97	40.4	9.28	0.03
	One day	17	7.1	26	10.8		
	Two days	48	20.0	31	12.9		
	More than two days	65	27.1	86	35.8		
BMI (kg/m^2)	Underweight (<18.5)	35	14.6	36	15.0	3.31	0.35
	Normal weight (18.5-24.9)	120	50.0	133	55.4		
	Overweight (25-29.9)	58	24.2	42	17.5		
	Obese (≥30)	27	11.2	29	12.1		

X^2:Chi-square test; FET: Fisher Exact Test; z: the Mann Whitney test; statistical significance was considered at P<0.05; BMI: Body Mass Index; SR: Saudi Rial; NBU: Northern Border University

Table 2: Relationship between eating habits and sociodemographic characteristics of students at health colleges, NBU

Characteristics		Inadequate eating habits (N.=107)		Adequate eating habits (N.=133)		X^2	P
		N.	%	N.	%		
Gender	Female	72	67.3	81	60.9	1.05	0.31
	Male	35	32.7	52	39.1		
Age (year)	<21	49	45.8	39	29.3	7.37	0.02
	21-23	37	34.6	65	48.9		
	≥23	21	19.6	29	21.8		
Academic year	First	19	17.8	14	10.5	8.40	0.21
	Second	17	15.9	13	9.8		
	Third	19	17.8	32	24.1		
	Fourth	25	23.4	38	28.6		
	Fifth	11	10.3	18	13.5		
	Sixth	5	4.8	10	7.5		
	Internship	11	10.3	8	6.0		
Marital status	Single	103	96.3	128	96.2	FET	1.00
	Married	4	3.7	5	3.8		
Place of residence	Owned	88	82.2	117	88.0	1.56	0.21
	Rented	19	17.8	16	12.0		
Living arrangement	Live alone	15	14.0	8	6.0	4.38	0.04
	Live with family	92	86.0	125	94.0		
Household monthly income (SR)	3,000-10,000	94	87.8	113	85.0	0.42	0.52
	>10,000	13	12.1	20	15.0		
Number of family members	≤5	21	19.6	17	12.8	3.39	0.18
	6-9	55	51.4	83	62.4		
	≥10	31	29.0	33	24.8		
Mothers' educational level	Not educated	9	8.4	9	6.8	3.76	0.29
	Low	18	16.8	12	9.0		
	Moderate	21	19.6	29	21.8		
	High	59	55.1	83	62.4		
Fathers' educational level	Not educated	5	4.7	6	4.5	FET	0.86
	Low	8	7.5	12	9.0		
	Moderate	35	32.7	37	27.8		
	High	59	55.1	78	58.6		
Separated parents	No	95	88.8	128	96.2	5.01	0.02
	Yes	12	11.2	5	3.8		
Working mother	No	58	54.2	59	44.4	2.30	0.13
	Yes	49	45.8	74	55.6		
Smoking	No	99	92.5	123	92.5	0.00	0.99
	Yes	8	7.5	10	7.5		
Playing sports/week	No	59	55.1	51	38.3	7.94	0.047
	One day	6	5.6	11	8.3		
	Two days	15	14.0	33	24.8		
	> two days	27	25.2	38	28.6		
BMI (kg/m^2)	Underweight (<18.5)	23	21.5	12	9.0	9.16	0.03
	Normal weight (18.5-24.9)	46	43.0	74	55.6		
	Overweight (25-29.9)	28	26.2	30	22.6		
	Obese (≥30)	10	9.3	17	12.8		

X^2:Chi-square test; FET: Fisher Exact Test; statistical significance was considered at $P<0.05$; BMI: Body Mass Index; SR: Saudi Rial, NBU: Northern Border University

.

Table 3: Relationship between eating habits and sociodemographic characteristics of students at non-health colleges, NBU

Characteristics		Inadequate eating habits (N.=99)		Adequate eating habits (N.=141)		X^2	P
		N.	%	N.	%		
Gender	Female	66	66.7	101	71.6	0.68	0.41
	Male	33	33.3	40	28.4		
Age (year)	<21	49	49.5	59	41.8	4.79	0.09
	21-23	28	28.3	59	41.8		
	≥23	22	22.2	23	16.3		
Academic year	First	15	15.1	21	14.9	4.54	0.34
	Second	26	26.3	31	22.0		
	Third	27	27.3	33	23.4		
	Fourth	22	22.2	48	34.0		
	Fifth	9	9.1	8	5.7		
Marital status	Single	92	92.9	128	90.8	FET	0.81
	Married	6	6.1	12	8.5		
	Divorced	1	1.0	1	0.7		
Place of residence	Owned	80	80.8	117	83.0	0.19	0.67
	Rented	19	19.2	24	17.0		
Living arrangement	Live alone	6	6.1	6	4.3	0.40	0.53
	Live with family	93	93.9	135	95.7		
Household monthly income (SR)	3,000-10,000	91	91.9	122	86.5	1.69	0.19
	>10,000	8	8.1	19	13.5		
Number of family members	≤5	14	14.1	21	14.9	0.04	0.98
	6-9	58	58.6	81	57.4		
	≥10	27	27.3	39	27.7		
Mothers' educational level	Not educated	7	7.1	13	9.2	2.56	0.46
	Low	21	21.2	24	17.0		
	Moderate	35	35.3	41	29.1		
	High	36	36.4	63	44.7		
Fathers' educational level	Not educated	8	8.1	13	9.2	3.51	0.32
	Low	7	7.1	17	12.1		
	Moderate	48	48.5	53	37.6		
	High	36	36.4	58	41.1		
Separated parents	No	86	86.9	135	95.7	6.28	0.01
	Yes	13	13.1	6	4.3		
Working mother	No	73	73.7	95	67.4	1.12	0.29
	Yes	26	26.3	46	32.6		
Smoking	No	88	88.9	132	93.6	1.70	0.19
	Yes	11	11.1	9	6.4		
Playing sports/week	No	54	54.5	43	30.5	16.23	0.001
	One day	11	11.1	15	10.6		
	Two days	11	11.1	20	14.2		
	> two days	23	23.2	63	44.7		
BMI (kg/m^2)	Underweight (<18.5)	17	17.2	19	13.5	4.36	0.22
	Normal weight (18.5-24.9)	47	47.5	86	61.0		
	Overweight (25-29.9)	21	21.2	21	14.9		
	Obese (≥30)	14	14.1	15	10.6		

X^2:Chi-square test; FET: Fisher Exact Test; statistical significance was considered at P<0.05; BMI: Body Mass Index; SR: Saudi Rial; NBU: Northern Border University

.

Table 4: Relationship between psychological factors and eating habits among students of health and non-health colleges, NBU

Psychological factors		Health colleges: Inadequate eating habits (N.=107) N.	%	Adequate eating habits (N.=133) N.	%	X^2	P	Non-health colleges: Inadequate eating habits (N.=99) N.	%	Adequate eating habits (N.=141) N.	%	X^2	P
Eating because of feeling lonely	Often	9	8.4	7	5.3	1.10	0.58	15	15.1	16	11.3	0.92	0.63
	Sometimes	41	38.3	56	42.1			36	36.4	50	35.5		
	Rarely	57	53.3	70	52.6			48	48.5	75	53.2		
feeling completely out of control when it comes to food	Often	11	10.3	13	9.8	0.22	0.90	11	11.1	10	7.1	9.79	0.007
	Sometimes	34	31.8	39	29.3			45	45.4	41	29.1		
	Rarely	62	57.9	81	60.9			43	43.4	90	63.8		
Eating so much until stomach hurts	Often	14	13.1	12	9.0	2.97	0.23	22	22.2	7	5.0	22.32	<0.001
	Sometimes	37	34.6	37	27.8			40	40.4	46	32.6		
	Rarely	56	52.3	84	63.2			37	37.4	88	62.4		
Eating because of feeling upset or nervous	Often	16	14.9	16	12.0	0.64	0.73	16	16.2	11	7.8	4.95	0.08
	Sometimes	33	30.8	46	34.6			38	38.4	51	36.2		
	Rarely	58	54.2	71	53.4			45	45.4	79	56.0		
Eating because of feeling bored	Often	23	21.5	25	18.8	0.44	0.80	20	20.2	20	14.2	3.24	0.20
	Sometimes	53	49.5	65	48.9			48	48.5	62	44.0		
	Rarely	31	29.0	43	32.3			31	31.3	59	41.8		
Eating because of feeling happy	Often	19	17.8	32	24.1	1.43	0.49	18	18.2	36	25.5	2.15	0.34
	Sometimes	55	51.4	62	46.6			52	52.5	63	44.7		
	Rarely	33	30.8	39	29.3			29	29.3	42	29.8		

X^2:Chi-square test; Statistical significance was considered at $P<0.05$; NBU: Northern Border University
.

Table 5: Logistic regression of adequate eating habits conditioned on significant risk factors among students of health and non-health colleges, NBU

Risk factors	Health colleges (N.=240)		Risk factors	Non-health colleges (N.=240)	
	OR (95% CI)	P		OR (95% CI)	P
**Separated parents**			**Separated parents**		
Yes vs. no	0.23(0.07-0.75)	0.01	Yes vs. no	0.19(0.06-0.60)	0.005
**Playing sports/week**			**Playing sports/week**		
No	1.00		No	1.00	
One day	4.22(1.22-14.63)	0.02	One day	2.20(0.84-5.78)	0.11
Two days	3.40(1.54-7.48)	0.002	Two days	1.82(0.75-4.40)	0.18
> Two days	2.23(1.12-4.44)	0.02	> Two days	3.61(1.81-7.20)	<0.001
**BMI (kg/m^2)**			**Eating so much until stomach hurts**		
Underweight (<18.5)	1.00				
Normal weight (18.5-24.9)	2.87(1.23-6.69)	0.01	Often	1.00	
Overweight (25-29.9)	1.86(0.72-4.77)	0.20	Sometimes	4.73(1.63-13.69)	0.004
Obese (≥30)	3.95(1.28-12.20)	0.02	Rarely	9.19(3.21-26.37)	<0.001
**Age (year)**			**Eating because of feeling happy**		
<21	1.00		Often	1.00	
21-23	2.58(1.33-4.99)	0.005	Sometimes	0.40(0.18-0.87)	0.02
≥23	2.02(0.92-4.45)	0.08	Rarely	0.59(0.25-1.38)	0.22
**Living arrangement**					
Live with family vs. live alone	3.04(1.10-8.41)	0.03			

OR: Odd's Ratio; 95% CI: 95% Confidence Interval; statistical significance was considered at P<0.05; NBU: Northern Border University.
