# Supplementary material for: Eating habits of students of health colleges and non-health colleges at the Northern Border University in the Kingdom of Saudi Arabia
Source: PLoS One. 2024 Oct 28;19(10):e0312750. doi: 10.1371/journal.pone.0312750 (PMC11516010; doi:10.1371/journal.pone.0312750)
Supplement: Attachment — Submitted filename: response to reviewers.docx [file pone.0312750.s004.docx]

**Responses to academic editor and reviewers’ comments**

**We sincerely appreciate the reviewers for their comments to improve this article. All responses are in red colour.**

**Areas for improvement :**

- STORBE checklist was attached under « Other » files.
- Study objectives were revised.
- Study limitations and potential bias were mentioned.
- Psychological factors were reported subjectively, as self-reported behaviors, and this was mentioned as a limitation of the study.
- Discussion of the results were compared with existing literature in Saudi arabia, other Arab countries and non-Arab countries and finding were justified.
- Conclusion and recommendation : a conclusion based on study findings was provided and recommendations to enhance healthy eating habits of university students through potential activities such as student activities, awareness campaigns, and academic courses were provided.

Ethical considerations : “This study was approved by the Local Committee of Bioethics (HAP-09-A-043) at NBU no. (27/44/H). Informed consent was obtained from all students prior to their participation. “

Response to reviewers’ comments:

1- **(page 8) frequent change in key words like eating habits/eating pattern. Can’t understand the reason**

Thank you for your comment, “eating patterns” was changed to “eating habits”.

2- **(page 9) Use key words what is in your title abstract, avoid changing keywords. Title says eating habits and introduction talk about healthy diet.**

Thank you, “A healthy diet is one that provides enough micronutrients…” was changed to “Healthy eating habits provide enough micronutrients….”

3- **(page 9) Abbreviation should be immediately after the full name**

Thank you, this was done

4- **(page 9) Reference 7 does not justify the claim in the introduction.**

Thank you, this reference was replaced by “Pan, A., Lin, X., Hemler, E., & Hu, F. B. (2018). Diet and Cardiovascular Disease: Advances and Challenges in Population-Based Studies. *Cell metabolism*, *27*(3), 489–496. https://doi.org/10.1016/j.cmet.2018.02.017”

5- **(page 9) Keyword changed**

Thank you, “eating behaviors” was changed to “eating habits”

6- **(page 10) Aim changed what has been mentioned in the abstract.**

Thank you, in the abstract, “This study aimed to determine and compare eating patterns between students at health and non-health colleges at Northern Border University (NBU), Saudi Arabia and to identify the relationship between eating patterns and sociodemographic and psychological characteristics of students.” was modified to “This study’s objectives were to identify and compare eating habits between students at health and non-health colleges at Northern Border University (NBU), Saudi Arabia and to determine the relationship between students’ eating habits and their sociodemographic, lifestyle, and psychological factors.”

7- **(page 10) Keyword changed**

Thank you, “eating patterns” was changed to “eating habits”.

8- **(page 11) How the response could be wrong? Why the author has used "wrong response" word?**

Thank you, “wrong responses” was changed to “non-healthy responses”.

9- **(page 12) if all the students of health college has been approached why more than half of the population data is from clinical years while opposite population distribution has been observed in non health college data? Author has already mentioned in the introduction that poor clinical duty related stress is one of the major contributing factor, then more clinical student student in the data may have biased the results.**

Thank you for your comment, for health colleges, 47.5% (n.=114) of students were from basic years (years1-3) and 52.5% (n.=126) were from clinical years (years 4-6 and the internship), which is not a significant difference (Z=1.55 and p=0.52), so this difference is unlikely to affect the results.

10- **(page 13) Justify how the significance level has been identified, while only change in the percentage has been presented in fig 1.**

Thank you, comparisons were carried out using the Chi-square test and statistical significance was considered at p<0.05, as mentioned in the Statistical analysis. “Categorical data were compared using the Chi-square test (x^2^) and the Fisher Exact Test (FET) as appropriate. ………….. Statistical significance was considered at P<0.05.” Also, here is the table showing details of Figure 1.

Supplementary Table 1: Comparisons of eating habits between students at health and non-health colleges

| Eating habits | | Non-health colleges  (N.=240) | | Health colleges  (N.=240) | | X^2^ | P |
| --- | --- | --- | --- | --- | --- | --- | --- |
|  |  | N. | % | N. | % |  |  |
| Do you have regular meals? | No | 146 | 60.8 | 151 | 62.9 | 0.22 | 0.64 |
|  | Yes | 94 | 39.2 | 89 | 37.1 |  |  |
| Do you have daily breakfast? | No | 132 | 55.0 | 144 | 60.0 | 1.23 | 0.27 |
|  | Yes | 108 | 45.0 | 96 | 40.0 |  |  |
| Frequency of daily meals | Less than three meals | 133 | 55.4 | 123 | 51.2 | 0.84 | 0.36 |
|  | Three or more times | 107 | 44.6 | 117 | 48.7 |  |  |
| Frequency of having snacks per week | Less than three times | 131 | 54.6 | 125 | 52.1 | 0.30 | 0.58 |
|  | Three or more times | 109 | 45.4 | 115 | 47.9 |  |  |
| Weekly consumption of vegetables and legumes | Less than three times | 151 | 62.9 | 145 | 60.4 | 0.32 | 0.57 |
|  | Three or more times | 89 | 37.1 | 95 | 39.6 |  |  |
| Weekly consumption of fruits | Less than three times | 201 | 83.7 | 204 | 85.0 | 0.14 | 0.71 |
|  | Three or more times | 39 | 16.2 | 36 | 15.0 |  |  |
| Weekly consumption of fried food | Less than three times | 152 | 63.3 | 137 | 57.1 | 1.96 | 0.16 |
|  | Three or more times | 88 | 36.7 | 103 | 42.9 |  |  |
| Consumption of fast food | Often | 71 | 29.6 | 68 | 28.3 | 0.09 | 0.95 |
|  | Sometimes | 157 | 65.4 | 160 | 66.7 |  |  |
|  | Rarely | 12 | 5.0 | 12 | 5.0 |  |  |
| Meals with family and friends | Daily | 113 | 47.1 | 121 | 50.4 | 0.53 | 0.46 |
|  | Not daily | 127 | 52.9 | 119 | 49.6 |  |  |
| Water intake | Less than two liters per day | 177 | 73.7 | 172 | 71.7 | 0.26 | 0.61 |
|  | Two or more liters per day | 63 | 26.2 | 68 | 28.3 |  |  |
| Type of food consumed | Mainly meat | 15 | 6.2 | 20 | 8.3 | 7.65 | 0.05 |
|  | Mainly vegetables | 13 | 5.4 | 4 | 1.7 |  |  |
|  | Carbohydrate | 70 | 29.2 | 86 | 35.8 |  |  |
|  | Variety of food in balance | 142 | 59.2 | 130 | 54.2 |  |  |

11- **(page 13) Justify how the significance level has been identified, while only change in the percentage has been presented in fig 2.**

Thank you, comparisons were carried out using the Chi-square test and statistical significance was considered at p<0.05, as mentioned in the Statistical analysis. “Categorical data were compared using the Chi-square test (x^2^) and the Fisher Exact Test (FET) as appropriate. ………….. Statistical significance was considered at P<0.05.” Also, here is the table showing details of Figure 2.

Supplementary Table 2: Comparisons of eating-related psychological factors between students at health and non-health colleges

| Psychological factors | | Non-health colleges  (N.=240) | | Health colleges  (N.=240) | | X^2^ | P |
| --- | --- | --- | --- | --- | --- | --- | --- |
|  |  | N. | % | N. | % |  |  |
| Do you eat because you are feeling lonely? | Often | 31 | 12.9 | 16 | 6.7 | 5.51 | 0.06 |
|  | Rarely | 123 | 51.2 | 127 | 52.9 |  |  |
|  | Sometimes | 86 | 35.8 | 97 | 40.4 |  |  |
| Do you feel completely out of control when it comes to food? | Often | 21 | 8.7 | 24 | 10.0 | 1.43 | 0.49 |
|  | Rarely | 133 | 55.4 | 143 | 59.6 |  |  |
|  | Sometimes | 86 | 35.8 | 73 | 30.4 |  |  |
| Do you eat so much until stomach hurts? | Often | 29 | 12.1 | 26 | 10.8 | 1.91 | 0.38 |
|  | Rarely | 125 | 52.1 | 140 | 58.3 |  |  |
|  | Sometimes | 86 | 35.8 | 74 | 30.8 |  |  |
| Do you eat because of feeling upset or nervous? | Often | 27 | 11.2 | 32 | 13.3 | 1.1 | 0.57 |
|  | Rarely | 124 | 51.7 | 129 | 53.7 |  |  |
|  | Sometimes | 89 | 37.1 | 79 | 32.9 |  |  |
| Do you eat because you are feeling bored? | Often | 40 | 16.7 | 48 | 20.0 | 2.57 | 0.28 |
|  | Rarely | 90 | 37.5 | 74 | 30.8 |  |  |
|  | Sometimes | 110 | 45.8 | 118 | 49.2 |  |  |
| Do you eat because you are feeling happy? | Often | 54 | 22.5 | 51 | 21.2 | 0.11 | 0.95 |
|  | Rarely | 71 | 29.6 | 72 | 30.0 |  |  |
|  | Sometimes | 115 | 47.9 | 117 | 48.7 |  |  |

12- **(page 14) Justification of findings should be written immediately after the claim or fining of this study followed by supporting evidence.**

Thank you, inadequate eating habits was defined in the Statistical analysis section as follows “The median dietary score was used to dichotomize dietary scores and logistic regression of adequate dietary habits (score≥4). For more justification “inadequate eating habits (dietary habits scores<4)” was added.

13- **(page 15) Every new paragraph in the Discussion should start with your findings rather than the findings of someone else**

Thank you, the sentence “Obesity has been linked to poor dietary habits. ^8^” was transferred to the end of the paragraph.

14- **(page 15) What is the need of mentioning prevalence of overweight/obesity in other arab countries here.**

Thank you, we wanted to compare our findings in Saudi Arabia, with previous findings in regional countries including the Gulf countries e.g., UAE and other Arab countries e.g., Jordan and Egypt.

15- **(page 15) Data given in this para is not gender specific while justification is gender based. Justify?**

Thank you, the overall conclusion from reference (35) supports our findings that practicing physical activity is low, and the reference add an explanation and a consequence for this finding. So, however our results were not gender specific because our study main objective was to compare eating habits not physical activity, but reference (35) supported our finding regarding physical activity.

16- **(page 15) How does this claim justify the objectives?**

Thank you, eating regular meals is a part of healthy eating habits, while our finding revealed that only the minority of students reported eating regular meals. For more clarification, the paragraph was modified into “Our study revealed that only 37.1% and 39.2% of students at health and non-health colleges, respectively reported eating regular meals, which was comparable to …”

17- **Generalized justification which lack scientific evidence.**

Thank you, the results of references 41 and 42 support this justification.

18- **Everywhere in the discussion data is compared with other countries. while scientific justification of the findings are missing.**

Thank you, this cross-sectional study aimed to compare and identify eating habits among university students. Comparing our results with previous studies in Saudi Arabia and other countries helps to identify differences in eating habits among students from different contexts. For justification the following sentence was added “Fast food is convenient, easily accessible, and often fits students’ busy schedules. Additionally, peer influence plays a significant role as eating at fast-food chains is a social activity among young people besides the high frequency of skipping breakfast among studied students. Also, fast food is marketed heavily towards teens and young people”
